# VGLL4 Selectively Represses YAP-Dependent Gene Induction and Tumorigenic Phenotypes in Breast Cancer

**DOI:** 10.1038/s41598-017-06227-7

**Published:** 2017-07-21

**Authors:** Yinglong Zhang, He Shen, Henry G. Withers, Nuo Yang, Kayla E. Denson, Ashley L. Mussell, Alexander Truskinovsky, Qingyu Fan, Irwin H. Gelman, Costa Frangou, Jianmin Zhang

**Affiliations:** 1 Department of Orthopedics, the First Affiliated Hospital of Chinese People’s liberate Army General Hospital, Beijing, 100048 China; 2 Department of Cancer Genetics & Genomics, Roswell Park Cancer Institute, Buffalo, NY 14263 USA; 3 Department of Pathology, Roswell Park Cancer Institute, Buffalo, NY 14263 USA; 4 Orthopedic Oncology Institute, Tangdu Hospital, Fourth Military Medical University, Xi’an, Shaanxi 710038 China

## Abstract

Members of the mammalian Vestigial-like (VGLL) family of transcriptional cofactors activate genes in response to a wide variety of environmental cues. Recently, VGLL proteins have been proposed to regulate key signaling networks involved in cancer development and progression. However, the biological and clinical significance of VGLL dysregulation in human breast cancer pathogenesis remains unknown. Here, we report that diminished VGLL4 expression, but not VGLL1-3, correlated with both shorter relapse-free survival and shorter disease-specific survival of cancer patients with different molecular subtypes of breast cancer. Additionally, we further demonstrate that overexpression of VGLL4 reduces breast cancer cell proliferation, migration, intravasation/extravasation potential, favors cell death, and suppresses tumor growth *in vivo*. Mechanistically, VGLL4 negatively regulates the TEAD1-YAP1 transcriptional complex and exerts its growth inhibitory control through its evolutionary conserved TDU2 domain at its C-terminus. The results suggest that VGLL4 is a candidate tumor suppressor gene which acts by selectively antagonizing YAP-dependent tumor growth. VGLL4 may be a promising therapeutic target in breast cancer.

## Introduction

Breast cancer is the second leading cause of cancer death in women. Despite improved treatment strategies, a major challenge is recurrent disease associated with resistance to treatment and intra-tumor heterogeneity^1^. While predictive biomarkers for response to systemic therapy could improve drug development efficiency, progress in identifying such markers has been slow. High-throughput sequencing tools for nucleic acid characterization now provide the unique opportunity to perform comprehensive analyses of all alterations in the cancer genomes^2^. However, our ability to systematically analyze this information and identify disease-relevant genes is limited. Thus, there is an urgent need to identify essential cancer driver events and related signaling pathways that can be exploited therapeutically.

Vestigial-like (VGLL) proteins, which include VGLL1-4, are cofactors for TEA domain-containing transcription factors (TEADs)^3^. Although TEAD transcription factors have been extensively studied concerning their essential role in normal development and human diseases such as cancer, little is known about the underlying mechanisms by which dysregulated VGLL1-4 genes exert their tumor-promoting activity^4^. Moreover, it is unclear whether VGLL proteins promote tumor growth *in vivo*. Nevertheless, several studies suggest that these proteins potentially play dual roles as either tumor suppressors in promoting cancer progression or oncogenes in tumor maintenance. For example, the VGLL1-TEAD transcription complex promotes anchorage-independent cell proliferation by up-regulating the expression of the proliferation-promoting gene IGFBP-5^5^. Furthermore, the inhibition of VGLL3 expression leads to a reduction in the proliferation and migration of soft tissue sarcoma lines *in vitro* ^6^. Conversely, in gastric and lung cancers, VGLL4 overexpression can suppress tumor growth *via* negatively regulating inhibitor of apoptosis proteins (IAPs)^7^.

In the present study, we sought to determine the role of VGLL proteins in breast cancer pathogenesis. By combining multi-genomic data from patient tumors with functional studies in breast cancer cell lines and tumor models, we report that VGLL4 functions as a novel suppressor of breast tumor growth and malignant progression. Low VGLL4 gene expression in clinical breast cancer specimens correlated with a poor patient prognosis. Consistent with these observations, ectopic VGLL4 expression in malignant breast cancer cell lines reduced cell proliferation, cell migration, and colony formation *in vitro* and tumor formation in xenograft mouse model. Mechanistically, we found that VGLL4 interacts with TEAD1 *via* its second TEAD-interacting domain (TDU2), selectively antagonizing the TEAD1-YAP1 transcriptional complex and, therefore, YAP-dependent tumor growth. Collectively, these results establish a clear role for VGLL4 in breast cancer and as such may have broad implications, both as a novel prognostic biomarker and a target for future therapeutic applications.

## Results

### Genomic analyses of VGLL1-4 in breast cancer specimens

The biological and clinical relevance of dysregulated VGLL expression in human breast cancer pathogenesis are unknown. To address these questions, we interrogated multi-dimensional cancer patient genomics datasets, using information readily available in The Cancer Genome Atlas (TCGA), which has enabled standard data collection procedures and results of extensive molecular profiling assays^8, 9^. We used somatic point mutation, copy-number alteration, and gene expression data from the TCGA Breast Invasive Carcinoma project^10^.

**Figure 1:**
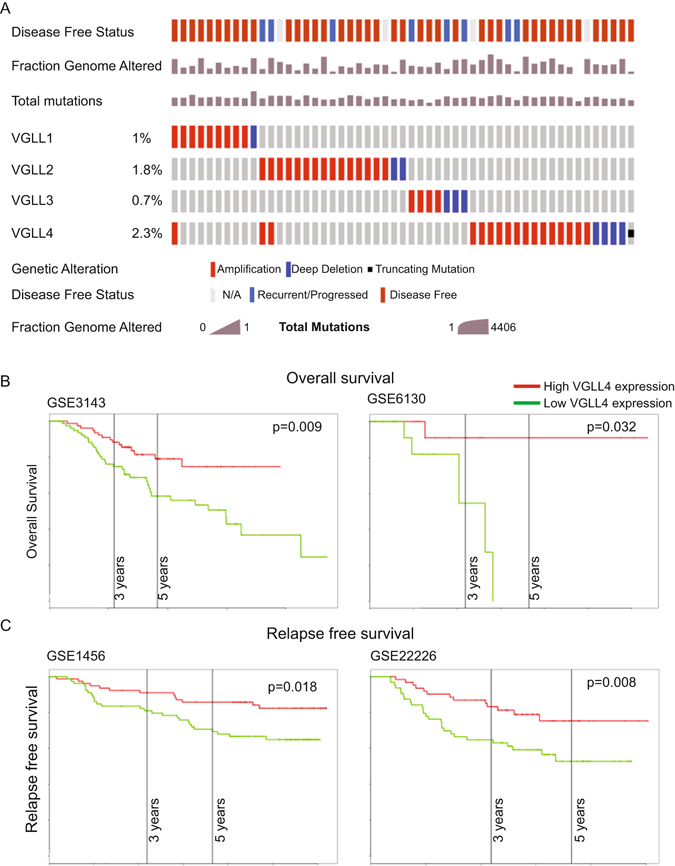
Coordinated analysis of VGLL1-4 mutation status and correlations with the genomic and clinical breast cancer features. (**A**) VGLL1-4 genetic alterations in 817 breast invasive carcinoma samples from TCGA. Copy number variation (CNV and mutation status normalized with MANCIE. Significantly mutated genes with frequent copy number amplifications (red) or deletions (blue) are shown. Average mutation rate is indicated. (**B**) Kaplan-Meier overall survival (OS) and (**C**) relapse-free survival (RFS) analysis of breast cancer patients using a median split of VGLL4 gene expression (KM-plotter). The Log Rank test was used to measure the statistical difference between the high and low VGLL4 groups for Kaplan-Meier curves. One-way ANOVA was used to measure the differences in VGLL4 expression in breast cancer patients of various subtypes. *X*-axis: follow-up time in years; *y*-axis: cumulative survival. Four independent patient data sets were used from the Gene Expression Omnibus (GSE3143, GSE6130, GSE1456 and GSE22226).

High-dimensional genomic data analysis is challenging due to systematic noise and biases in high-throughput (HT) experiments^11^. To overcome these challenges, we used MANCIE (matrix analysis and normalization by concordant information enhancement); an integrative computational method that can conduct data normalization and bias correction for high-dimensional genomic data integration^12^. We applied MANCIE on TCGA datasets consisting of luminal A, luminal B, HER2-enriched, basal-like and claudin-low breast cancer subtypes; each of which have unique biological and prognostic features. We first investigated the somatic mutation spectrum and copy number aberrations for VGLL1-4 in the context of PAM50 breast subtype^13^, but neither copy number, mutation type or mutation frequency shared mRNA correlative patterns (Fig. 1A and Figure S1a). We next examined VGLL1-4 gene expression in breast cancer samples, including a subset of tumor-matched normal tissue samples. Within breast cancer samples, VGLL1-4 expression patterns varied considerably across different histologic subtypes (data not shown). Furthermore, we found that VGLL1-4 expression did not correlate with tumor grade and showed no capacity to stratify breast cancer patients into good *versus* poor outcome groups (Figure S1b).

### VGLL4 Gene Expression Level Correlates with Clinicopathological Features of Breast Cancer Patients

Breast cancer is not a single disease but is instead comprises subtypes that have distinct histopathological features, genetic and genomic variability, and diverse prognostic outcomes^14^. Moreover, clinical tumor samples contain a heterogeneous mixture of cell types that can confound relationships between transcriptome levels and clinical correlates^15^. To overcome this limitation, we examined VGLL1-4 expression levels in a panel of 30 breast cancer cell lines that mirror most of the important genomic and resulting transcriptional abnormalities found in primary breast tumors and have been extensively used in mechanistic studies^16^. VGLL1-4 gene expression levels closely resembled those in TCGA patient samples. Furthermore, comparisons of relative VGLL gene expression revealed VGLL3 and VGLL4 were abundantly expressed across different breast cancer subtypes (Figure S2a).

We next sought to determine the clinical significance of VGLL1-4 expression in breast cancer. The correlation between the relative mRNA expression level of these genes and clinicopathological features were examined by univariate Kaplan-Meier analyses. Remarkably, a positive association between decreased VGLL4 gene expression and lower median overall survival and relapse-free survival within five years of diagnosis was observed using several independent patient data sets (Fig. 1B and C)^17–20^. Correspondingly, a multivariate analysis of the prognosis factors with a Cox proportional hazards model confirmed that low VGLL4 expression was an independent predictor of poor survival in breast cancer and remained significant when adjusting for other prognostic factors such as age, gender, tumor size or histological type (Supplemental Table 1 and data not shown). Taken together, VGLL1-4 germline or somatic mutations are extremely rare. Furthermore, dysregulation of VGLL4 expression, but not VGLL1-3, is commonly observed in patients with different molecular subtypes of breast cancer and correlated with a poor patient prognosis (Fig. 1).

### VGLL4 Inhibits *In vitro* Breast Cancer Growth

**Figure 2:**
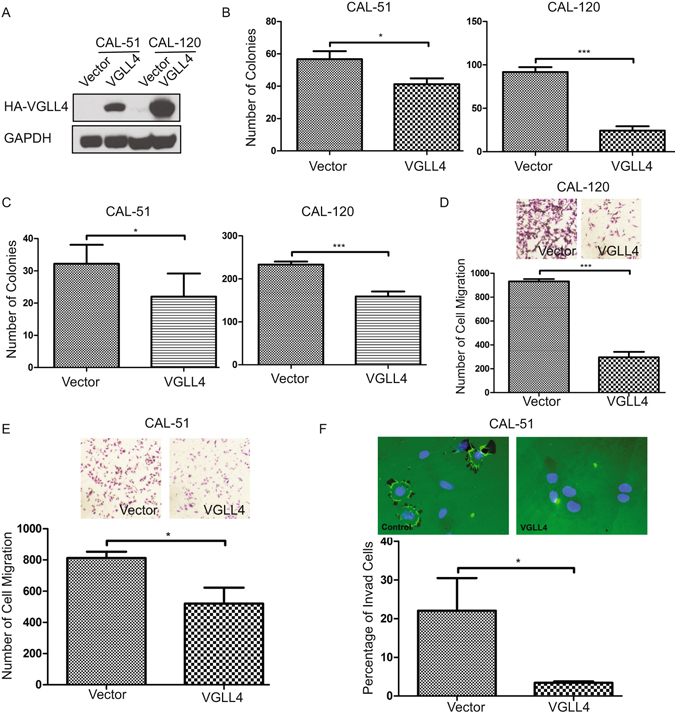
VGLL4 overexpression inhibits the proliferation, colony-formation and transformation abilities of breast cancer cells *in vitro*. (**A**) Immunoblot of ectopic VGLL4 expression in the CAL-51 and CAL-120 cells. Quantification of colony-formation (**B**) and anchorage-independent growth in soft agar (**C**) of vector- or VGLL4-transduced CAL-51 and CAL-120 cells. Representative migration images and quantification of cell migration of vector- or VGLL4-transduced CAL-120 (**D**) and CAL-51 cells (**E**). (**F**) Representative invasion images and quantification of cell invasion of vector- or VGLL4-transduced CAL-51 cells. (*P < 0.05, **P < 0.01, ***P < 0.001).

Given clinical data linking aberrant VGLL4 expression to breast cancer development and progression, we next explored the functional relevance of VGLL4 expression on breast cancer cell proliferation and migration *in vitro* and tumor growth *in vivo*. To this end, we overexpressed VGLL4 in two breast cancer cell lines, CAL-51 and CAL-120 (Fig. 2A), that express relatively low but detectable levels of VGLL4. Ectopic VGLL4 expression inhibited proliferation of these two cell lines (Figure S3a) and inhibited colony formation in 2D culture (Figs 2B and S3b) and anchorage-independent cell growth in soft agar (Figs 2C and S3c). In contrast to mammary carcinoma cells, VGLL4 overexpression in either immortalized MCF10A cells derived from benign proliferative breast tissue or primary human mammary epithelial cells did not dramatically influence *in vitro* growth or tumorigenic potential (data not shown).

**Figure 3:**
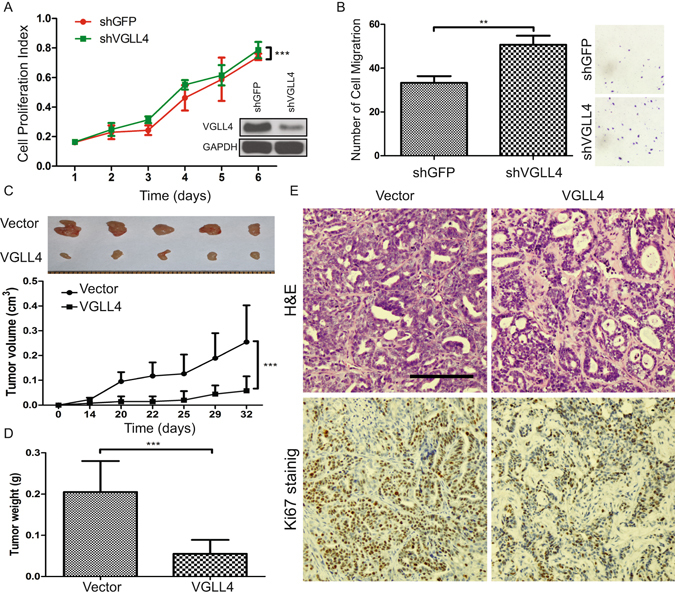
Knockdown of VGLL4 promotes cell proliferation and VGLL4 overexpression suppresses breast tumor growth *in vivo*. (**A**) Cell proliferation by the MTT assay for shGFP- or shVGLL4-transduced T47D cells (inset: VGLL4 knockdown efficiency demonstrated by immunoblot). (**B**) Migration images and quantification of T47D cell transduced with shGFP or shVGLL4. Overexpression of VGLL4 in CAL-51 cells dramatically reduces the tumor growth potential (**C**) (top panel: representative tumor images), tumor weight (**D**). (**E**) H&E and Ki67 immunohistochemistry (IHC) staining of xenograft tumors. (Scale bar = 100 µm) (**P < 0.01, ***P < 0.001).

Acquisition of invasive cell behavior underlies tumor progression and metastasis^21^. In culture, the invasive behavior of cells is often monitored by evaluating their ability to move through a layer of extracellular matrix (ECM), in a Transwell Chamber. We used a Transwell migration assay and a gelatin invasion assay to determine the effects of VGLL4 on breast cancer cell migration and invasion. For both CAL-51 and CAL-120 cell lines, compared with control cells, cells overexpressing VGLL4 exhibited decreased cell migration (Fig. 2D,E). Especially, for CAL-51 cells, VGLL4 overexpression dramatically inhibited cell invasion by >80% (Fig. 2F). Finally, to extend our observations, we used shRNA targeting VGLL4 to efficiently knockdown endogenous VGLL4 in T47D, a breast cancer cell line that expresses high levels of VGLL4. Inhibition of VGLL4 markedly enhanced cell proliferation and cell migration (Fig. 3A,B).

### VGLL4 Suppresses Breast Cancer Progression in a Xenograft Mouse Model

To test whether VGLL4 modulates tumorigenesis *in vivo*, we subcutaneously injected control or VGLL4-overexpressing CAL-51 cells into severe combined immunodeficiency (SCID) mice. Both tumor growth rate and tumor size were dramatically reduced for VGLL4-overexpressing cells compared with control cells (Fig. 3C,D). Morphologically, cancerous cells are characterized by large nuclei, irregular size and shape, prominent nucleoli^22^. Consistent with previous reports, histological analyses demonstrated that CAL51 tumors were moderately differentiated and showed several features characteristic of human breast cancer, including microvascular proliferation, focal micropapillary architecture and areas of tumor necrosis bordered by dense palisades of viable tumor cells (necrosis with pseudopalisading) (Fig. 3E top left panel). However, ectopic VGLL4 expression altered growth characteristics of primary, subcutaneous tumors. Slides were stained with hematoxylin and eosin (H&E) and detected foci were less eosinophilic than the surrounding tissue and composed of smaller cells with smaller nuclei, cytoplasmic vacuolization and frequent apoptotic cells (Fig. 3E top right panel). Immunohistochemistry (IHC) analysis confirmed that VGLL4-overexpressing tumors exhibited decreased cell proliferation by using the proliferation marker Ki-67 (Fig. 3E lower panel). Together, our results suggest that VGLL4 regulates several important characteristics of tumorigenesis, functionally suppresses breast cancer growth, migration, invasion *in vitro* and tumor formation *in vivo*.

### Transcriptome Profiling Identifies Key VGLL4-regulated Pathways

**Figure 4:**
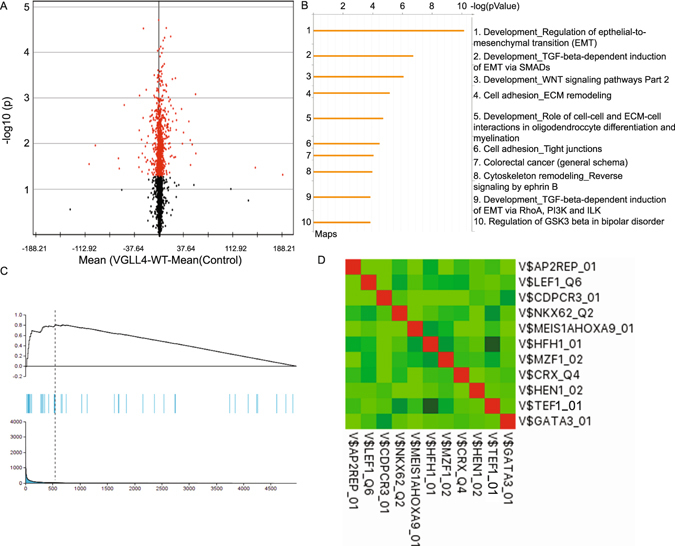
VGLL4-regulated gene expression. (**A**) Volcano plot displaying the −log10 of the P values from modified T-test in terms of the log2 fold change for VGLL4 overexpressing cells. The selected genes have significantly different expression values (P ≤ 10−15–P ≤ 10−3). (**B**) GeneGo MetaCore data analysis of VGLL4-induced genes. Columns correspond to canonical pathways names −log(p value), percentage of genes detected in this pathway and molecules in this pathway. The top 10 pathways are summarized, additional information provided in Supplemental Table 2. (**C**) GSEA result analysis to assess enrichment of the YAP gene expression signature. NES, normalized enrichment score; FDR q-val, false discovery rate q-value (the probability that a gene set with a given NES represents a false-positive finding). (**D**) The cross-correlation coefficients of transcription factor’s contribution value (TFCV) score among 10 selected highest transcription factor binding site candidate models using all known position weight matrices.

Tumor suppressor genes involved in cancer have sustained ‘loss-of- function’ defects that inactivate their function and expression^23^. To gain insight into the role of VGLL4 as a candidate tumor suppressor in breast cancer, we compared the global transcription profile of VGLL4-overexpressing breast cancer cells to control cells. Specifically, to determine differentially expressed genes (DEG’s) between control vector and VGLL4 overexpressing cells, we conducted a paired analysis that used the criteria of a false discovery rate (FDR)-corrected P value ≤ 0.05 and a fold-change threshold of ≥1.5. We identified 591 DEG’s (Fig. 4A) with 226 upregulated genes and 365 downregulated genes in control cells, as compared with VGLL4 overexpressing cells. Next, to explore the relevance of gene expression changes following overexpression of VGLL4, we performed network and pathway analyses of the DEG’s using the Metacore Suite^24^. This approach identified several pathways that are important for human breast cancer including epithelial-to-mesenchymal transition (EMT), cell adhesion, TGF-β signaling, WNT signaling and blood vessel morphogenesis (Fig. 4B & Supplemental Table 2). Interestingly, our analysis also revealed a significant enrichment (nominal *P* values ≤ 0.05) for genes activated by YAP (Fig. 4C). We also used the Database for Annotation, Visualization, and Integrated Diseases (DAVID)^25^ to identify cellular processes that were enriched in VGLL4 overexpressed breast cancer cells. DAVID is a functional annotation tool used to interrogate Gene ID’s; using >40 annotation categories including gene ontology terms, protein-protein interactions, and biological pathways. Developmental processes, cell differentiation, and locomotion were significantly enriched processes with P-values of 6.050E-40, 6.960E-36, and 6.811E-32, respectively (Supplemental Table 3).

VGLL4 encodes for a cofactor that modulates the effects of transcription factors, but does not possess intrinsic DNA binding activity. We hypothesized that a list of genes, differentially regulated between control and VGLL4 overexpressing cells, would contain enriched transcription binding sites within their promoter regions. To test this hypothesis, we created a ranked list of target genes and scanned all differentially expressed promoter regions with the TRAP predictor algorithm^26^. We chose the TRAP approach since it avoids the artificial separation between binding and non-binding sites but instead calculates the binding probability of a given transcription factor to all sites in the sequence based on a biophysical model. The binding affinity of ~700 TFs, represented by position weight matrices (PWMs), in the Eukaryotic Promoter Database (EPD) to all human promoters was subsequently calculated. We identified several enriched transcription factor binding modules with the TEF1 (TEAD1) matrix among the top PWMs for the VGLL4 overexpression dataset (Fig. 4D). Correspondingly, we identified TEAD1 as the primary TEAD family member expressed in breast cancer cells (Figure S2).

### Tumor Potential is Strongly Correlated with YAP Activity

**Figure 5:**
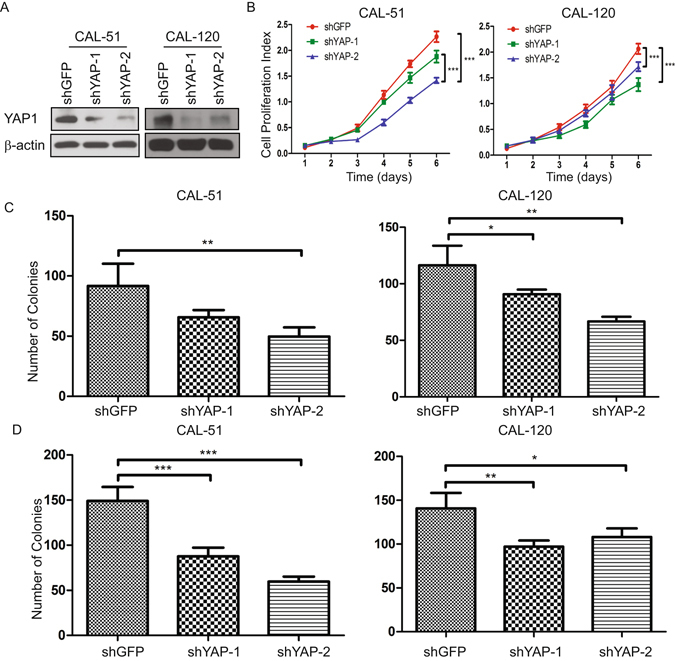
Knockdown of YAP inhibits breast cancer cell proliferation. (**A**) YAP knockdown efficiency in CAL-51 and CAL-120 cells by immunoblot. (**B**) Cell proliferation by the MTT assay for shGFP- or shYAP-transduced CAL-51 and CAL-120 cells. Quantification of colony-formation (**C**) and anchorage-independent growth in soft agar (**D**) for shGFP- or shYAP-transduced CAL-51 and CAL-120 cells. (*P < 0.05, **P < 0.01, ***P < 0.001).

EMT has been suggested as a mechanism by which immotile cancer cells acquire a more invasive and motile phenotype^27^. Furthermore, hyperactive YAP has previously been shown to induce EMT and the TEAD family transcription factors are essential in mediating YAP-dependent gene expression^28, 29^. With this background in mind, we used an RNAi approach to perturb endogenous YAP expression in CAL-51 and CAL-120 cells. YAP knockdown inhibited breast cancer cell proliferation (Fig. 5A,B), cell growth by 2D colony formation (Figs 5C and S4a), anchorage-independent growth in soft agar (Figs 5D and S4b).

Next, to test the predictive value of our model we validated our findings in a panel of human breast tumor-derived cell lines, with annotated cancer-driving genetic lesions promoting the oncogenic state and associated with dependencies that are specific to these lesions^30^. As predicted, VGLL4 had a dramatic suppressive growth effect on CAL-120 and T47D cell lines (YAP-hyper-activated breast cancer cell lines). Conversely, VGLL4 had a negligible growth effect on HCC1143, DU4475, MDA-MB-468, BT-474, MDA-MB-134-VI and MDA-MB-453 cell lines, which are dependent on AKT, BRAF, EGFR, ERRB2, FGFR1and PIK3CA oncogenes, respectively (Figure S5). Taken together, our data demonstrate that VGLL4 suppresses aberrant proliferation in breast tumors by selective inhibition of the YAP onco-protein. More importantly, these findings also suggest that VGLL4 may play complex and context-dependent roles in the regulation of breast cancer cell survival and death, a question that currently remains unexplored.

### VGLL4 Negatively Regulates YAP-TEAD1 Complex Transactivation

**Figure 6:**
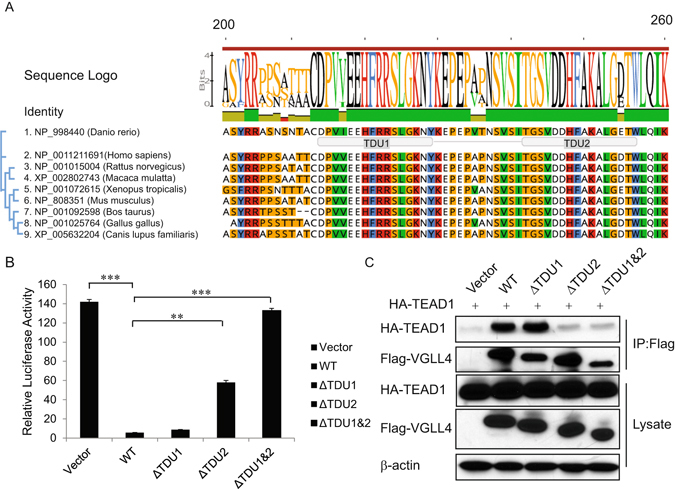
VGLL4 interacts with TEAD1 through the TDU2 domain. (**A**) Image of evolutionary conservation of VGLL4 and schematic representation of TDU1 and TDU2 domains. (**B**) VGLL4-WT or VGLL4-ΔTDU1 completely inhibits the TEAD response element reporter activity, whereas VGLL4-ΔTDU2 or VGLL4-ΔTDU1&2 fail to do so. (**C**) VGLL4 interacts with TEAD1 through its TDU2 domain. Co-immunoprecipitation of TEAD1 with the Flag-VGLL4 immunoprecipitation only observed in HEK293T cells co-transfected w/ TEAD1 and WT- or ΔTDU1-VGLL4 but not ΔTDU2- or ΔTDU1ΔTDU2-VGLL4.

VGLL4 contains two evolutionary conserved TDU domains and this feature distinguishes VGLL4 from other VGLL proteins (Fig. 6A). To assess the importance of the TEAD-binding domains of VGLL4, namely the tandem TDU domains, first, to determine whether VGLL4 inhibits TEAD activity, we performed a luciferase reporter assay using constructs containing the TEAD canonical response element^31^ to assess wild-type (WT) VGLL4 and VGLL4 with the deletion of one or both TDU domains (Fig. 6B). Overexpression of VGLL4 or VGLL4 with a TDU1 deletion (ΔTDU1) completely abolished luciferase activity associated with the TEAD response element; in contrast, VGLL4 with TDU2 deleted or TDU1 and TDU2 deleted (ΔTDU2 or ΔTDU1&2) failed to block this activity. To confirm the existence of TEAD1/VGLL4 complex, we examined the physical interaction of TEAD1 with VGLL4-WT and TDU deletion mutants. Interestingly, Co-IP assay in CAL51 cells showed that TEAD1 and VGLL4 co-precipitated but VGLL4-ΔTDU2 and VGLL4-ΔTDU1&2 could not bind to TEAD1 (Fig. 6C). Intriguingly, prior research has indicated that VGLL4 preferentially interacts with TEAD1 *via* the TDU1 domain^32^.

### VGLL4 TDU2 Domain is Sufficient and Necessary to Inhibit YAP Activity

**Figure 7:**
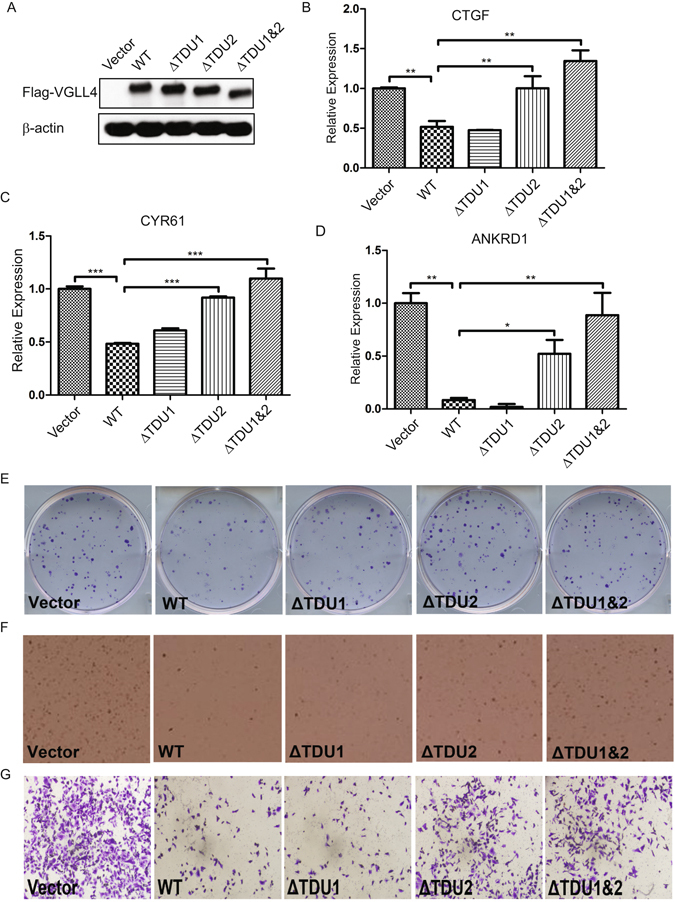
TDU2 domain deletion rescues the effect of VGLL4 suppression on cell proliferation and colony-formation *in vitro*. (**A**) Immunoblot of vector, WT or mutant VGLL4 expression in CAL-51 cells. Real-time qPCR measured expression levels of CTGF (**B**), CYR-61 (**C**) and ANKRD1 (**D**) for vector- or VGLL4-transduced CAL-51 cells. Representative images of colony-formation assay (**E**), anchorage-independent growth in soft agar (**F**) and cell migration (**G**) for vector, WT or mutant VGLL4 transduced CAL-51 cells. (*P < 0.05, **P < 0.01, ***P < 0.001).

To determine the functional significance of VGLL4 TDU domains, we compared the genome-wide expression profiles of CAL-51 cells transduced with VGLL4-WT, VGLL4-ΔTDU1, VGLL4-ΔTDU2 or VGLL4-ΔTDU1&2. Although the expression levels of most genes did not markedly change, differences for the expression levels of ~1200 genes were established by the use DESeq2 algorithm and Benjamin-Hochberg FDR based correction^33^. We generated a “heat map” by performing a hierarchical clustering analysis of DEGs across VGLL4 mutant cell lines (Figure S6a). Venn diagram representation analysis for understanding distribution of up and downregulated genes clearly showed gene signatures that are shared in a domain-specific manner (Figure S6b). VGLL4-WT and VGLL4-ΔTDU1 significantly inhibited the expression of the YAP target genes. In contrast, ΔTDU2 and ΔTDU1&2 failed to produce these effects (Figure S6c). We independently confirmed these results, using qRT-PCR. VGLL4-WT and VGLL4-ΔTDU1 inhibited the expression of YAP target genes CTGF and CYR61 by >60% (Fig. 7A–C) and completely inhibited ANKRD1 gene expression (Fig. 7D). In contrast, ΔTDU2 and ΔTDU1&2 failed to produce these effects (Fig. 7A–D).

### The VGLL4 TDU2 Domain is Sufficient to Inhibit YAP-dependent Tumorigenic Phenotypes in Selected Breast Cancer Subtypes

Anchorage-independent growth capability is an important characteristic of transformed cells, while decreased clonogenic potential is typically associated with the loss of invasion capabilities in tumor cells. VGLL4-WT and VGLL4-ΔTDU1 inhibited colony formation in 2D culture (Figs 7E and S7a), anchorage-independent growth in soft agar (Figs 7F and S7b) and cell migration (Figs 7G and S7c), in contrast, VGLL4-ΔTDU2 or VGLL4-ΔTDU1&2 failed to do so. In summary, these data demonstrate that VGLL4 negatively regulates the TEAD1-YAP1 transcriptional complex and exerts its growth inhibitory control through its evolutionary conserved TDU2 domain at its C-terminus.

## Discussion

The VGLL family of transcriptional coactivators plays important roles in the regulation of embryonic development, cell growth, and survival in response to diverse signals. Deregulation of VGLL1-4 gene function has been implicated in the pathogenesis of several human cancers, including lung, gastric cancer, and esophageal squamous cell carcinoma^4, 6^. However, little is known about the underlying molecular mechanisms by which these proteins exert their tumor-promoting activity. We present here both clinical and experimental evidence indicating that VGLL4 is a candidate tumor suppressor gene that operates by restraining oncogenic YAP-dependent responses promoting breast cancer progression. Importantly, the following evidence supports this conclusion: (1) VGLL4 gene expression was predictive of survival outcome of patients with breast cancer; (2) VGLL4 regulates several hallmarks of cancer - serving a critical function to inhibit cancer cell proliferation and survival *in vitro* and tumor growth *in vivo*; (3) overexpression of VGLL4 led to reduced migration and invasion potential suggesting that VGLL4 suppresses aggressive cancer behavior; (4) VGLL4 induces its anticancer inhibitory activity and its direct antitumor effects by selective modulation of YAP-dependent transcription.

The Hippo pathway is evolutionally conserved and regulates diverse cellular processes, including cell survival, proliferation, differentiation, organ size and tissue homeostasis^34^. Conversely, rewiring of the Hippo signaling network and its downstream target YAP exerts a significant impact on cancer development and progression^35^. Although Hippo pathway activities are known to be altered in human cancers, very few somatic and germline mutations of Hippo signaling components have been described so far^36, 37^. However, the YAP gene locus is amplified in several cancer subtypes^38^, while overexpressed or hyperactivated YAP has been reported in several cancer types^39–41^. Accordingly, the clinical significance of Hippo pathway makes it a highly attractive target for the development of novel cancer drugs. Furthermore, because core Hippo components are unaffected by genetic aberrations, reactivation of this pathway in cancer cells might restore the proper inhibition of oncogenic YAP.

Since all upstream Hippo pathway regulators converge upon YAP nuclear localization and transcriptional responses, pharmacological inhibition of YAP activity represents an effective anticancer strategy against human cancers caused by abnormal Hippo signaling. To this end, removing YAP from the nucleus or sequestration in the cytoplasm is theoretically the most efficient way to suppress its action regardless of its dependency on the Hippo signaling cascade. Unfortunately, upstream regulators that specifically promote YAP activity are typically non-enzymes, such as scaffolding, regulatory or structural proteins, and their activities are dependent on protein-protein interactions (PPIs). Unlike common druggable targets (i.e. G-protein coupled receptors, nuclear receptors, ion channels or enzymes), it has been difficult to identify small molecules that compete with the binding of an intracellular protein partner and can serve as therapeutic candidates.

Currently, the only pre-clinical lead compound targeting a cancer-driving PPI in Hippo signaling comes from studies addressing the YAP/TAZ interaction with the TEAD transcription factors^42^. In the present study, we clearly show VGLL4 regulates several important characteristics of tumorigenesis and metastasis. Mechanistically, VGLL4 interacts with TEAD1 both physically and functionally to inhibit TEAD1 transcriptional activity. The blockade of transcription factors interactions can be especially attractive in targeting cellular pathways that promote oncogenic transformation and typically involve multiple signaling proteins that ultimately converge on a much smaller set of oncogenic transcription factors. Synthetic molecules that mimic these domains could disrupt protein-protein contacts, thereby inhibiting the formation of multiprotein complexes. Besides, this approach may display the added advantage of reduced toxicity compared to targeting upstream signaling molecules are endowed with pleiotropic functions such as G-protein coupled receptors that have been reported to regulate LATS1/2 kinases and alter YAP phosphorylation and function^43^.

Notably, our study indicates that the tumor-inhibiting functions of VGLL4 are mediated *via* its TDU2 domain. To this end, we rationalize that mimics of VGLL4 or VGLL4-TDU1 may become promising drugs for delaying tumor progression. Consistent with this notion, a peptide mimicking VGLL4 function was recently reported to inhibit tumor development in a *Helicobacter pylori* mouse model of gastric cancer^44, 45^. This novel peptide could also present a promising option to inhibit YAP-TEAD-driven transcription in breast cancer. Nevertheless, additional studies are necessary to further explore the genetic variations underlying the association of these proteins with breast cancer.

In breast cancer, YAP interacts with TEADs to promote multiple processes such as proliferation, transformation, migration and invasion, which are necessary for tumorigenesis. Given its similar binding ability to TEADs, VGLL4 may represent a highly promising suppressor of YAP oncogenic activity and supported by several published studies. For example, in a mutagenic screen, VGLL4 was a gene identified as a potential candidate tumor suppressor in human pancreatic cancer, and VGLL4 expression was significantly associated with patient survival^46^. It was subsequently reported that VGLL4 could inhibit tumor progression Refs 47,48, and function as a tumor suppressor by negatively regulating the activity of the YAP-TEAD complex in lung and gastric cancer^4, 45^. Finally, YAP can induce miR130 expression to effectively repress the activity of VGLL4 and thereby amplify YAP signaling^49^. Taken together, these studies are consistent with the findings of the current study and further highlight the important role VGLL4 plays in restraining the oncogenic potential of YAP in breast cancer.

Breast cancer is a complex disease that involves a sequence of gene-environment interactions in a progressive process that cannot occur without dysfunctions in multiple systems^50, 51^. A major challenge in breast cancer research is the identification of cellular targets whose inhibitions selectively impair the growth of cancer cells while sparing normal cells. The results described here, suggest that pharmacological modulation of the VGLL4 signaling axis may represent a selective therapeutic strategy to inhibit YAP-induced tumorigenesis. Importantly, our observation that normal or non-transformed breast epithelial cells do not exhibit similar changes in cell growth or survival after ectopic expression of VGLL4 suggest that VGLL4 represents a unique class of cancer-specific growth-arresting and apoptosis-inducing genes, that may prove efficacious for the targeted therapy of breast cancer. Furthermore, our study provides a new conceptual framework for further understanding YAP-Hippo pathway function in breast cancer. Future studies are required to address the possibility of targeting VGLL4-YAP in breast cancer as a means of selectively modulating genes under the control of TEAD transcription factors.

## Methods

### Cell culture and plasmids

Human breast cell lines HCC1143, DU4475, MDA-MB-468, BT-474, MDA-MB-134-VI, AU565 and MDA-MB-453 and T47D, were purchased from ATCC (Manassas, VA). CAL-51 and CAL-120 cell lines were a kind gift from Dr. Toru Ouchi (Roswell Park Cancer Institute, NY). Cells were grown in DMEM medium (Corning Cellgro, NY), supplemented with 20% fetal bovine serum (FBS), penicillin (100 units/ml), streptomycin (100 µg/ml) under the 5% CO_2_ culture condition at 37 °C. VGLL4-WT was PCR amplified and inserted into the pBABE retroviral expression vector. VGLL4-ΔTUD1, ΔTUD2, and ΔTUD1&2 expression vectors were kindly provided by Dr. Ji^4^.

### Cell proliferation assay

CAL-51 control cells and VGLL4-overexpression cells (CAL-51-pBABE or CAL-51-pBABE-VGLL4) were seeded (~3000 cells per well) into 96-well plates. After culturing for 24 h, one plate was taken out, and 10 μL of MTT solution (5 mg/mL) was added to each well. After 3 hours incubation at 37 °C, the supernatant was removed, and 100 uL of MTT Solvent was added to each well. Then after 15 min incubation under dark, the absorbance was measured at 570 nm with a plate reader (Thermo Fisher Scientific; MA). This procedure was repeated every day until the 6th plate. The experiment was also performed for CAL-120 control cells and VGLL4-overexpression cells (CAL-120-pBABE or CAL-120-pBABE-VGLL4) with a density of 1500 cells per well.

### Colony-formation assay

CAL-51-pBABE, CAL-51-pBABE-VGLL4, CAL-120-pBABE, CAL-120-pBABE-VGLL4 cells were seeded separately into 6-well plates at a density of ~200 cells per well in triplicate. After culture in the incubator for 10 days, the plates were removed, and washed with 2 mL PBS. Plates were fixed with 1 mL 4% polyoxymethylene each well for 30 min and stained with 0.1% crystal violet solution (Sigma-Aldrich, MO) for 15 min. Then, after washing with PBS for 3 times, the colonies of each cell line were imaged and counted for statistical analysis.

### Soft agar colony formation assay

The soft agar colony formation assay is used to detect cellular anchorage-independent growth *in vitro*, a trait of transformation ability. Briefly, 2% agar was melted and incubated in the water bath at 37 °C. Then the agar was diluted with complete culture media to get the 0.5% agar, which was added into 6-well plate (2 mL per well). Then the plates were placed at 4 °C for 30 min and 37 °C for another 30 min. During this period, the cells were counted and suspended in 0.4% agar. After the 0.5% agar beds had been ready, 1.5 mL 0.4% agar containing 4.5 × 10^4^ were layered onto the 0.5% agar bed in each well. After incubation at 4 °C for 30 min, the plates were cultured at 37 °C incubator for ~3 weeks with supplementing 1 mL 0.4% agar every week. Finally, the colonies were stained with 0.02% iodonitrotetrazolium chloride solution (Sigma-Aldrich, MO) and photographed for counting.

### Transwell cell migration assay

Transwell inserts with an 8 µm pore polycarbonate membrane (Falcon, NY) were used for the migration assay. 800 μL of the complete culture media were added to the lower chambers. 1 × 10^5^ cells suspended in 300 μL complete culture media were seeded upper chambers. After 24 h of culture, the inserts were washed with PBS and swiped with cotton swabs to remove the cells remained on the inner surface of the membranes. Then the inserts were fixed with 4% paraformaldehyde for 30 minutes and stained with 0.1% crystal violet solution overnight. The next day, after washing with PBS migrated cells were imaged and counted under a light microscope from five random fields at a magnification of 100x.

### Gelatin cell invasion assay

Coverslips were washed clean and sterilized in advance and put into 6-well plates. Next day, 50 μL of 1 mg/mL Oregon green 488-gelatin (Life Technologies, MA) was diluted with 200 μL of cold 2% sucrose solution to make the 0.2 mg/mL gelatin solution, and then warmed in a water bath at 37 °C. Then the coverslips were all coated with the gelatin solution. After incubating each coverslip with 200 μL, 0.5% ice-cold glutaraldehyde in PBS, the plates were placed at 4 °C for 15 min. Coverslips were washed with PBS and incubated with 5 mg/mL sodium borohydride in PBS for 3 min. After washing with PBS for 3 times, sterilizing with 70% alcohol for 20 min and washing with PBS for another 3 times, the coverslips were incubated with serum-free culture media for 1 hour at 37 °C. Then 1 × 10^5^ cells were seeded into each well and cultured at 37 °C for 24 h. The next day, coverslips were washed with PBS and fixed with 1 mL 4% paraformaldehyde for 15 minutes. After washing with PBS again, the coverslips were incubated with 1 mL 0.05% Triton-X 100 for 10 min. After washing with PBS, the coverslips were mounted onto slides with Prolong Gold anti-fade reagent with DAPI (Invitrogen, MA). After drying the slides for 1–2 days, the coverslips were observed and imaged using a fluorescence microscope. All the procedures were performed in the dark and at room temperature.

### Quantitative real-time PCR (qRT-PCR)

Total RNA was extracted using Trizol Reagent (Life Technologies, MA) according to the manufacturer’s protocol. cDNA synthesis and quantitative real-time PCR was performed as previously described^52^. GAPDH was used as the internal control. The primer sequences were as follows:

VGLL4-F: 5′-GTGTCTTCCAACTTCCCTACAT-3′;

VGLL4-R: 5′-GCGTACGAGGAAGCGTATAAA-3′;

CTGF-F: 5′-GGAAATGCTGCGAGGAGTGG-3′;

CTGF-R: 5′-GAACAGGCGCTCCACTCTGTG-3′;

CYR61-F: 5′-CACACCAAGGGGCTGGAATG-3′

CYR61-R: 5′-CCCGTTTTGGTAGATTCTGG-3′

ANKRD1-F: 5′-GCCAAAGACAGAGAAGGAGATAC-3′

ANKRD1-R: 5′-GAGATCCGCGCCATACATAAT-3′

GAPDH-F: 5′-GTGAAGGTCGGAGTCAACGG-3′

GAPDH-R: 5′-GAGGTCAATGAAGGGGTCATTG-3′

All measurements were performed in triplicate (minimum, n = 3).

### Western blotting

The cell lysates were collected using RIPA buffer (Boston Bio-Products; MA) supplemented with Protease and Phosphatase Inhibitors (Thermo Scientific; MA). Briefly, sample proteins (30 or 40 µg) were separated by SDS-PAGE electrophoresis and then transferred to PVDF membranes (EMDMillipore; MA). After blocking with 5% BSA or non-fat milk for 1 h, the membranes were incubated with primary antibody overnight at 4 °C. The next day, the membranes were incubated with anti-rabbit, rat or mouse secondary antibody (Bio-Rad; CA) for 1 h; Finally the detection was performed using ECL Plus Western Blotting Detection Reagents (GE Healthcare; PA). anti-VGLL4 and anti-Flag M2 antibodies (Sigma-Aldrich; MO); anti-YAP antibody (Santa Cruz; CA); anti-Tubulin, GAPDH and β-actin antibodies (Ubiquitin-Proteasome Biotechnologies; CO); anti-HA antibody (Roche Diagnostics; IN).

### Small hairpin RNA (shRNA)

The shVGLL4 construct^4^ was generated in the pLKO.1 vector at the AgeI/EcoRI sites:

sense: 5′-CCGGGAGCCTGGGCAAGAATTACAACTCGAGTTGTAATTCTTGCCCAGGCTCTTTTTG-3′;

antisense: 5′-AATTCAAAAAGAGCCTGGGCAAGAATTACAACTCGAGTTGTAATTCTTGCCCAGGCTC-3′.

### *In vivo* tumor growth assay

The female NOD/SCID mice of 6–8 weeks old were obtained from the Roswell Park Cancer Institute (RPCI). The care and use of animals were performed under the rules provided by Declaration of Helsinki and approved by the Institutional Animal Care and Use Committee of the Roswell Park Cancer Institute (Buffalo, NY). Briefly, 1 × 10^6^ cells (CAL-51-pBABE or CAL-51-pBABE-VGLL4) were injected subcutaneously and observed twice a week after the injection. After ~1 month, the mice were sacrificed, and the tumors were weighed and processed for image analysis.

### TCGA Breast Cancer data

Copy number, mutation, transcriptome and clinical data were obtained from the TCGA Data Portal (https://tcga-data.nci.nih.gov/tcga/tcgaHome2.jsp). To eliminate the heterogeneity introduced by different sequencing platforms, we only downloaded those data in the category of UNC (IlluminaHiSeq_ RNASeqV2). Two classes of phenotypes were used “primary tumor” and “solid tissue normal”, namely only those samples in the clinical category of “primary tumor” or “solid tissue normal” were used for this study.

### Strand-oriented RNA-Sequencing

For each tissue, a strand-oriented RNA library was prepared to preserve information about which DNA strand was the original template during the synthesis of transcripts, thus offering strand orientation for detection of antisense transcription and providing information about regulatory relationships.

Cytoplasmatic rRNA removal was performed for each total RNA sample using the Ribo-Zero rRNA Removal Kit (Epicentre, Madison, WI, USA). The rRNA-depleted RNA was used to prepare the stranded-oriented RNA-seq library using the TruSeq Stranded Total RNA Sample Prep Kit (Illumina, San Diego, CA, USA), according to the manufacturer’s instructions. Briefly, each RNA was chemically fragmented before the random priming reverse transcription reaction for first strand cDNA generation. The fragmentation step resulted in an RNA-seq library including inserts ranging in size from approximately 100–400 bp. During the second strand synthesis, dUTP was incorporated in place of dTTP, thus preventing amplification of this strand during the subsequent PCR step and retaining strand information. cDNA libraries were sequenced on the Illumina NextSeq platform (Illumina Inc.). Paired-end reads of 75 nt were generated for each fragment.

### Alignment of RNA-Seq reads

Base calls were made using the Illumina CASAVA pipeline encoded in Phred 33. RNA-Seq reads in FASTQ format were inspected using FASTQC program. Adaptors and low quality regions (phred cutoff of 20) were trimmed using TrimGalore, excluding reads with final length less than 35 bases. Gene abundance estimation for all transcripts was calculated using the RNA-Seq by Expectation-Maximization (RSEM) method.

RSEM quantities calculated during de novo assembly were used to assess differential transcript expression separately using the DESeq2 Bioconductor software package.

Reference human transcriptome was obtained from the iGenomes repository (http://support.illumina.com/sequencing/sequencing_software/igenome.html), and annotations for rRNA genes were downloaded from UCSC genome browser selecting the RepeatMask table. Transcripts were considered differentially expressed at a p-value < 0.05 following a Benjamini and Hochberg false discovery rate (FDR) adjustment of 5% (0.05). Resulting DEGs were compared between analyses using VENNPLEX.

### Statistical analysis

Pearson χ2-test was used to test the correlation between VGLL protein levels and clinical parameters, Ki67 index. Relapse -free survival (RFS) and overall survival (OS) described the survival function for both Kaplan-Meier survival analyses and Cox proportional hazard univariate and multivariate regression analyses. Statistical significance was set at p < 0.05.

### Data availability

All sequencing data produced in the present work have been submitted to NCBI Gene Expression Omnibus (GEO).

## Electronic supplementary material

Supplementary information

Supplementary Table 2

Supplementary Table 3
