# Supplementary material for: VGLL4 Selectively Represses YAP-Dependent Gene Induction and Tumorigenic Phenotypes in Breast Cancer
Source: Sci Rep. 2017 Jul 21;7:6190. doi: 10.1038/s41598-017-06227-7 (PMC5522454; doi:10.1038/s41598-017-06227-7)
Supplement: Supplementary file 1 — Supplementary information [file 41598_2017_6227_MOESM1_ESM.pdf]

VGLL4 Selectively Represses YAP-Dependent Gene Induction and Tumorigenic Phenotypes in Breast Cancer

Yinglong Zhang [1, 2#], He Shen [2#], Henry G. Withers [2], Nuo Yang [2], Kayla E. Denson [2], Ashley L. Mussell [2], Alexander Truskinovsky [3], Qingyu Fan [4], Irwin H. Gelman [2], Costa Frangou [2,*] and Jianmin Zhang [2,*]

[1] Department of Orthopedics, the First Affiliated Hospital of Chinese People's liberate Army General Hospital, Beijing, 100048, China

[2] Department of Cancer Genetics & Genomics,

[3] Department of Pathology, Roswell Park Cancer Institute, Buffalo, NY 14263, USA

[4] Orthopedic Oncology Institute, Tangdu Hospital, Fourth Military Medical University, Xi'an, Shaanxi 710038, China

*To whom correspondence should be addressed. Email: gus.frangou@RoswellPark.org; jianmin.zhang@roswellpark.org

[#] these authors contributed equally to this work

Supplementary information

Supplementary figure legends

Supplemental Table1. Univariate Cox Regression analysis of VGLL4 gene expression and breast cancer patient survival.

Supplemental Table 2. VGLL4 regulated pathways.

Supplemental Table 3. VGLL4 regulated GO process.

Supplemental Figure Legends

Figure S1. VGLL1-4 gene expression and correlations with mutation status and clinical breast cancer features.

(**a**) Comparison of VGLL1-4 gene expression, mutation frequency and copy number alterations.

(**b**) The relationship between VGLL1-4 gene expression and tumor stage.

Figure S2. Heat map analysis of VGLL1-4 and TEAD1-4 gene expression in a panel of breast cancer cell lines.

(**a**)VGLL1-4 relative quantities by qPCR.

(**b**) TEAD1-4 relative quantities by qPCR from a panel of breast cancer cell lines. Each row shows expression of a single gene and colored according to the fold change relative to the median. Cell line names are labeled on the right.

Figure S3. VGLL4 overexpression inhibits the proliferation, colony-formation and transformation abilities of breast cancer cells *in vitro*.

(a) Cell proliferation by the MTT assay for vector- or VGLL4-transduced CAL-51 and CAL-120 cells.

Representative images of colony formation (**b**) and anchorage-independent growth in soft agar (**c**) for vector- or VGLL4-transduced CAL-51 and CAL-120 cells.

Figure S4. Knockdown of YAP inhibits breast cancer cell proliferation.

Representative images of colony formation (**a**), anchorage-independent growth in soft agar (**b**) for shGFP- or shYAP-transduced CAL-51 and CAL-120 cells.

Figure S5. Transcriptome profiling of the VGLL4 domain mutants in CAL-51 cells.

(**a**)Heat map generated from the*VGLL4* transcriptome data reflecting gene expression values. Fold-change relative to control cells rendered in a blue–white–yellow pseudo color scheme for all genes with $P < 0.005$ and $FC_{log2} > 1.5$ or $FC_{log2} < -1.5$.

(**b**) Venn diagram drawn by the VennPlex program summarizing gene overlap between VGLL4 and TDU domain mutants. The numbers in the 4-set Venn diagram indicate the output set orders in the resultant extracted data panel. To indicate the polarity of numerical regulation of each factor within each set or intersection, a two or three-way key system was employed. The number of factors unique to a set, with a positive regulation polarity, is identified with an italic numeral. The number of factors unique to a set, with a negative regulation polarity, is identified with an underlined numeral. The number of factors common between multiple sets is indicated with a red colored numeral.

(**c**) Heat map for GSEA result analysis to assess enrichment of YAP gene expression signature (CORDENONSI_YAP_CONSERVED_SIGNATURE). Bar color reflects gene expression fold change from blue (low), white (medium) to yellow (high).

Figure S6. Cellular viability of breast cancer cells expressing TET-inducible VGLL4.

(**a**) List of a panel of breast cancer cell lines with different genetic lesions.

(**b**) Cellular viability measured 1, 5 and 9 days post VGLL4 induction by doxycycline in a panel of breast cancer cell lines using CellTiter-Glo (Promega). All error bars represent SD, n=3. (*** $p<0.001$)

Figure S7. TDU2 domain deletion rescues the effect of VGLL4 suppression on cell proliferation and colony-formation *in vitro*.

Quantification of colony-formation assay (**a**), anchorage-independent growth in soft agar (**b**) and cell migration (**c**) for vector, WT or mutant VGLL4 transduced CAL-51 cells. (*P < 0.05, **P<0.01, ***P<0.001)

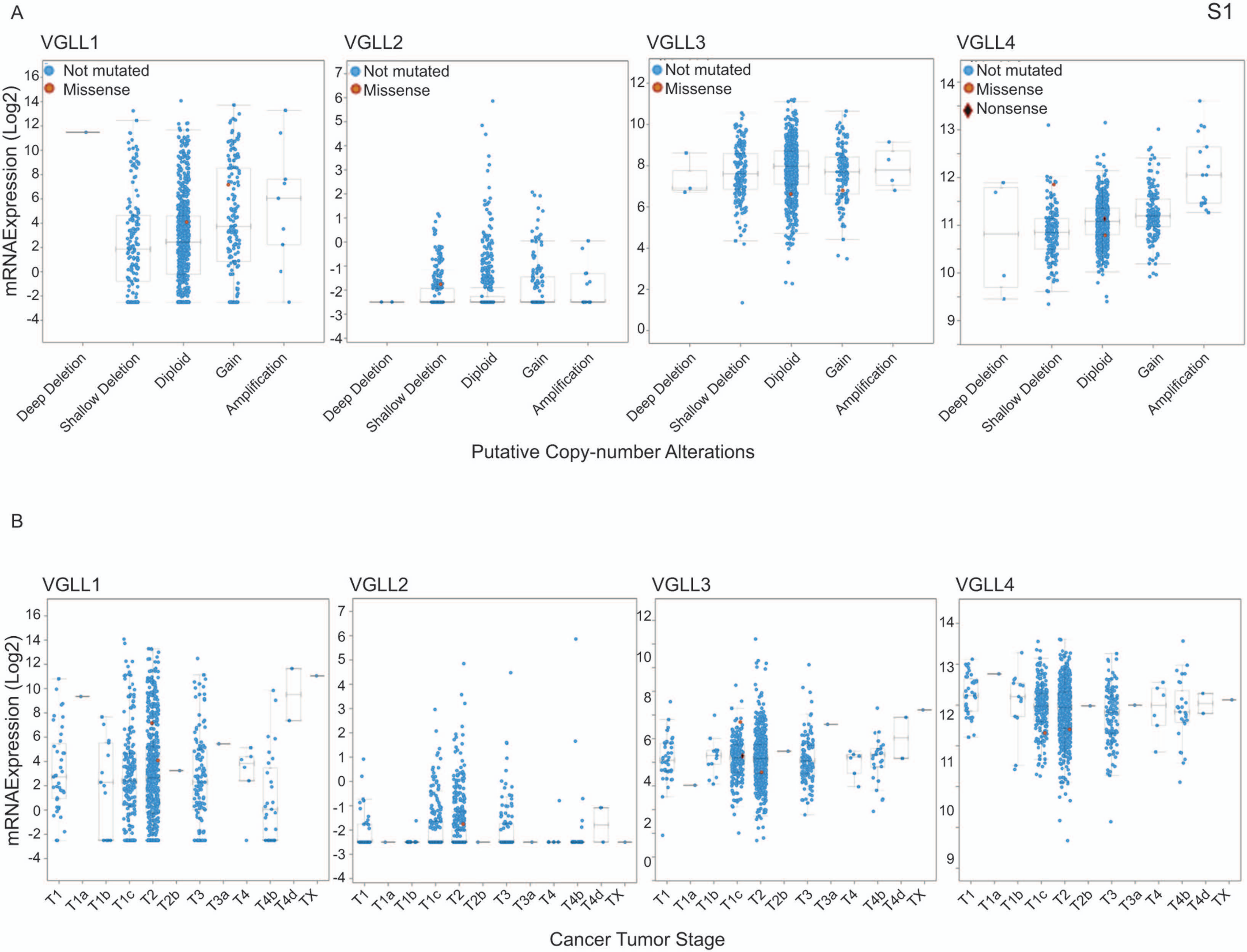

S1
A
VGLL1
VGLL2
VGLL3
VGLL4
Not mutated
Missense
Nonsense
mRNAExpression (Log2)
Deep Deletion
Shallow Deletion
Diploid
Gain
Amplification
Putative Copy-number Alterations
B
VGLL1
VGLL2
VGLL3
VGLL4
mRNAExpression (Log2)
T1
T1a
T1b
T1c
T2
T2b
T3
T3a
T4
T4b
T4d
TX
Cancer Tumor Stage

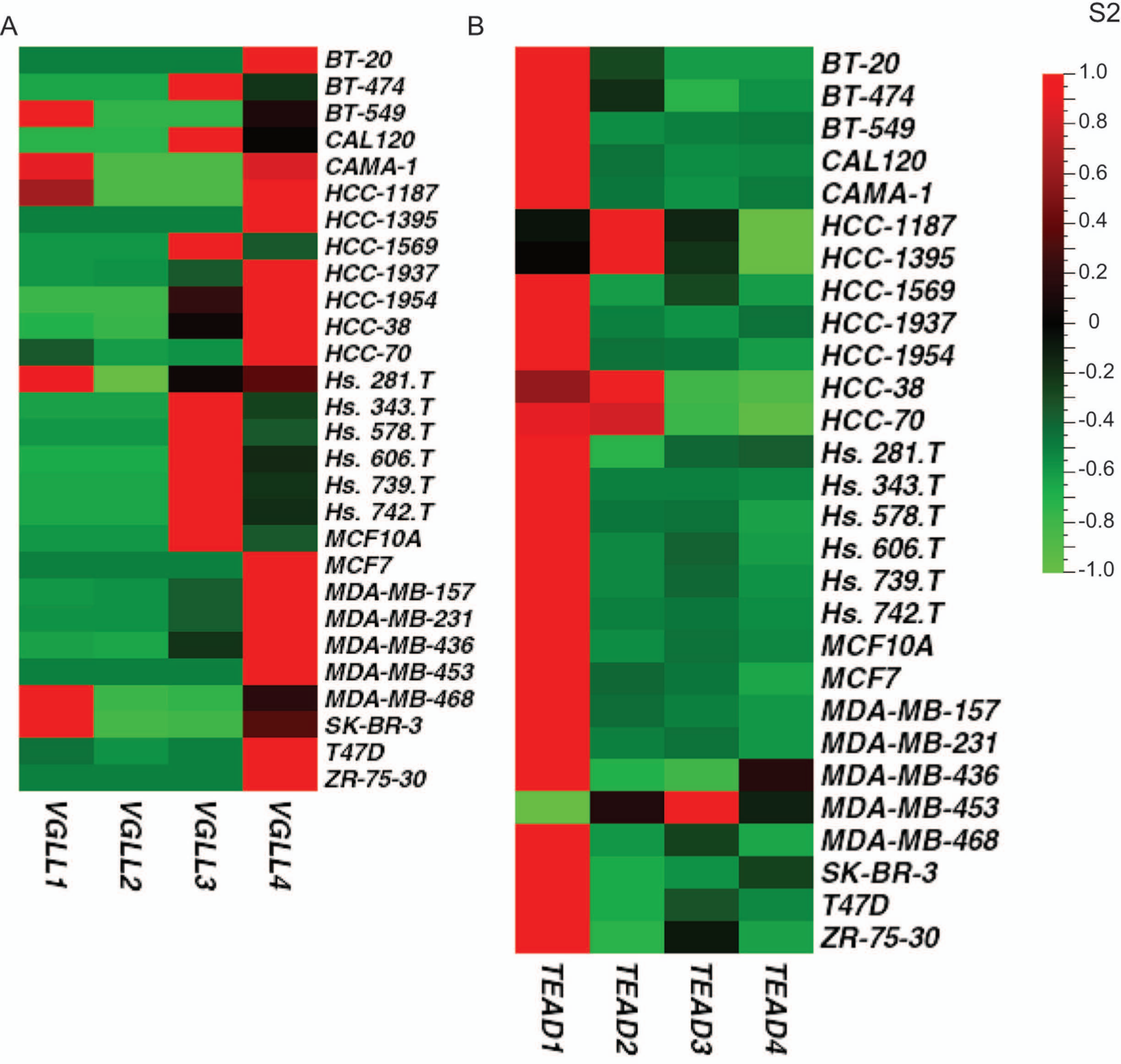
S2
A
BT-20
BT-474
BT-549
CAL120
CAMA-1
HCC-1187
HCC-1395
HCC-1569
HCC-1937
HCC-1954
HCC-38
HCC-70
Hs. 281.T
Hs. 343.T
Hs. 578.T
Hs. 606.T
Hs. 739.T
Hs. 742.T
MCF10A
MCF7
MDA-MB-157
MDA-MB-231
MDA-MB-436
MDA-MB-453
MDA-MB-468
SK-BR-3
T47D
ZR-75-30
VGLL1
VGLL2
VGLL3
VGLL4
B
BT-20
BT-474
BT-549
CAL120
CAMA-1
HCC-1187
HCC-1395
HCC-1569
HCC-1937
HCC-1954
HCC-38
HCC-70
Hs. 281.T
Hs. 343.T
Hs. 578.T
Hs. 606.T
Hs. 739.T
Hs. 742.T
MCF10A
MCF7
MDA-MB-157
MDA-MB-231
MDA-MB-436
MDA-MB-453
MDA-MB-468
SK-BR-3
T47D
ZR-75-30
TEAD1
TEAD2
TEAD3
TEAD4
1.0
0.8
0.6
0.4
0.2
0
-0.2
-0.4
-0.6
-0.8
-1.0

S3

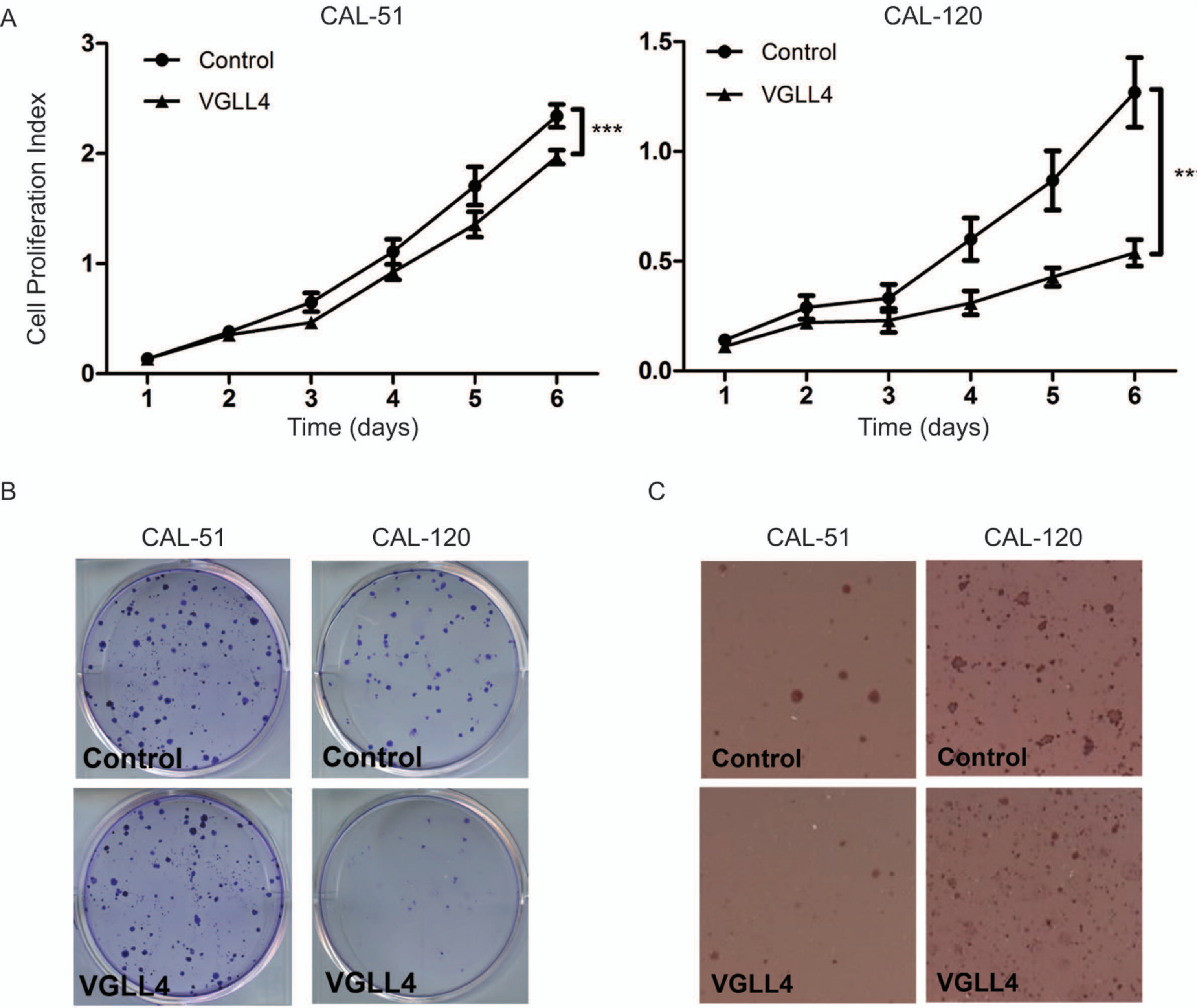
A
CAL-51
Control
VGLL4
***
Cell Proliferation Index
0
1
2
3
1
2
3
4
5
6
Time (days)
CAL-120
Control
VGLL4
***
0.0
0.5
1.0
1.5
1
2
3
4
5
6
Time (days)
B
CAL-51
CAL-120
Control
Control
VGLL4
VGLL4
C
CAL-51
CAL-120
Control
Control
VGLL4
VGLL4

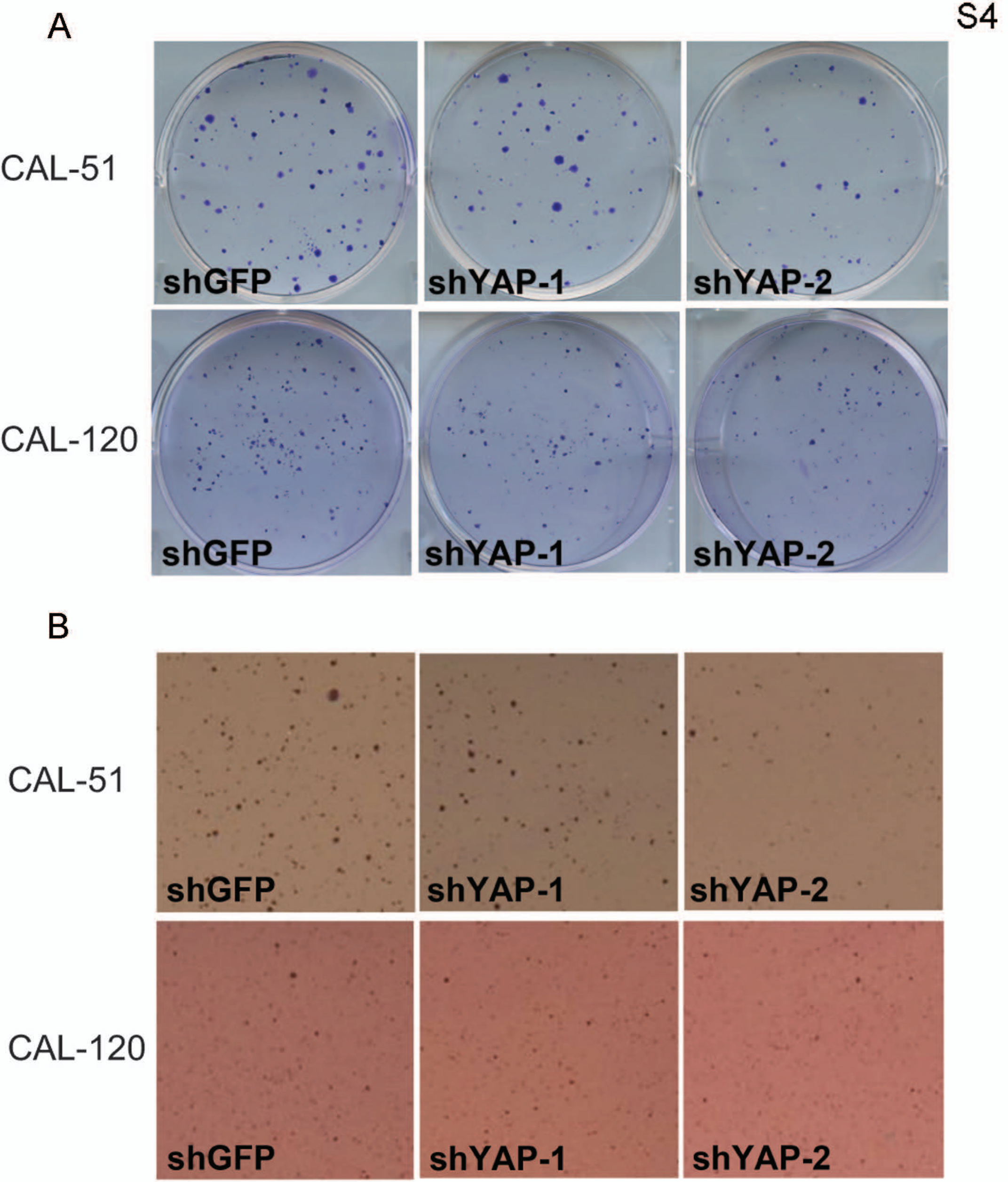
S4
A
CAL-51
shGFP
shYAP-1
shYAP-2
CAL-120
shGFP
shYAP-1
shYAP-2
B
CAL-51
shGFP
shYAP-1
shYAP-2
CAL-120
shGFP
shYAP-1
shYAP-2

A

Cell line	Genetic alterations	Tumor source
CAL-120	YAP hyperactivation	Breast
T47D	YAP hyperactivation	Breast
HCC1143	AKT1 amplification	Breast
DU4475	BRAF mutation (V600E)	Breast
MDA-MB-468	EGFR amplification	Breast
BT-474	ERBB2 amplification	Breast
MDA-MB-134-VI	FGFR1 amplification	Breast
MDA-MB-453	PIK3CA mutation (H1047R)	Breast

B

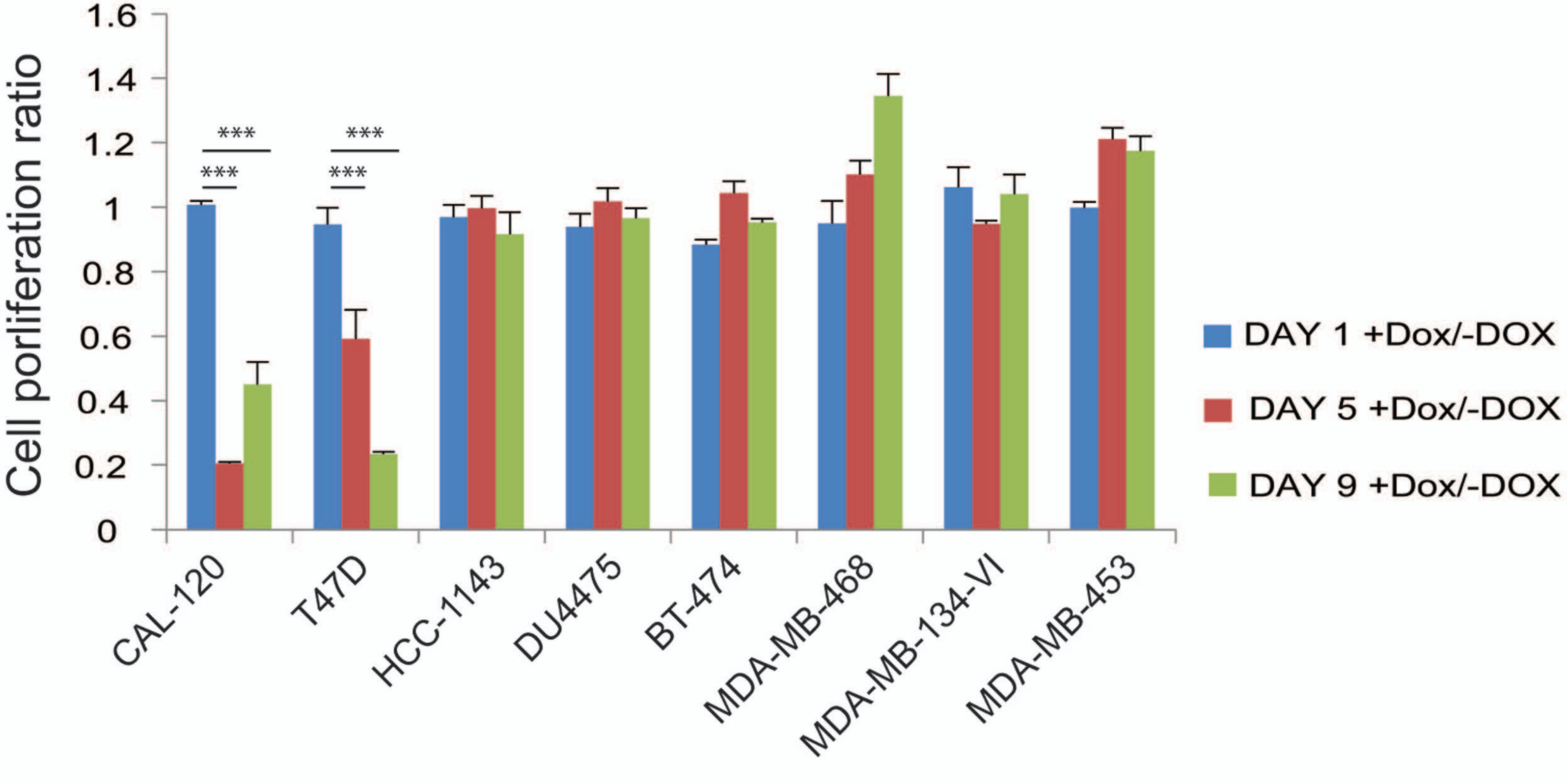

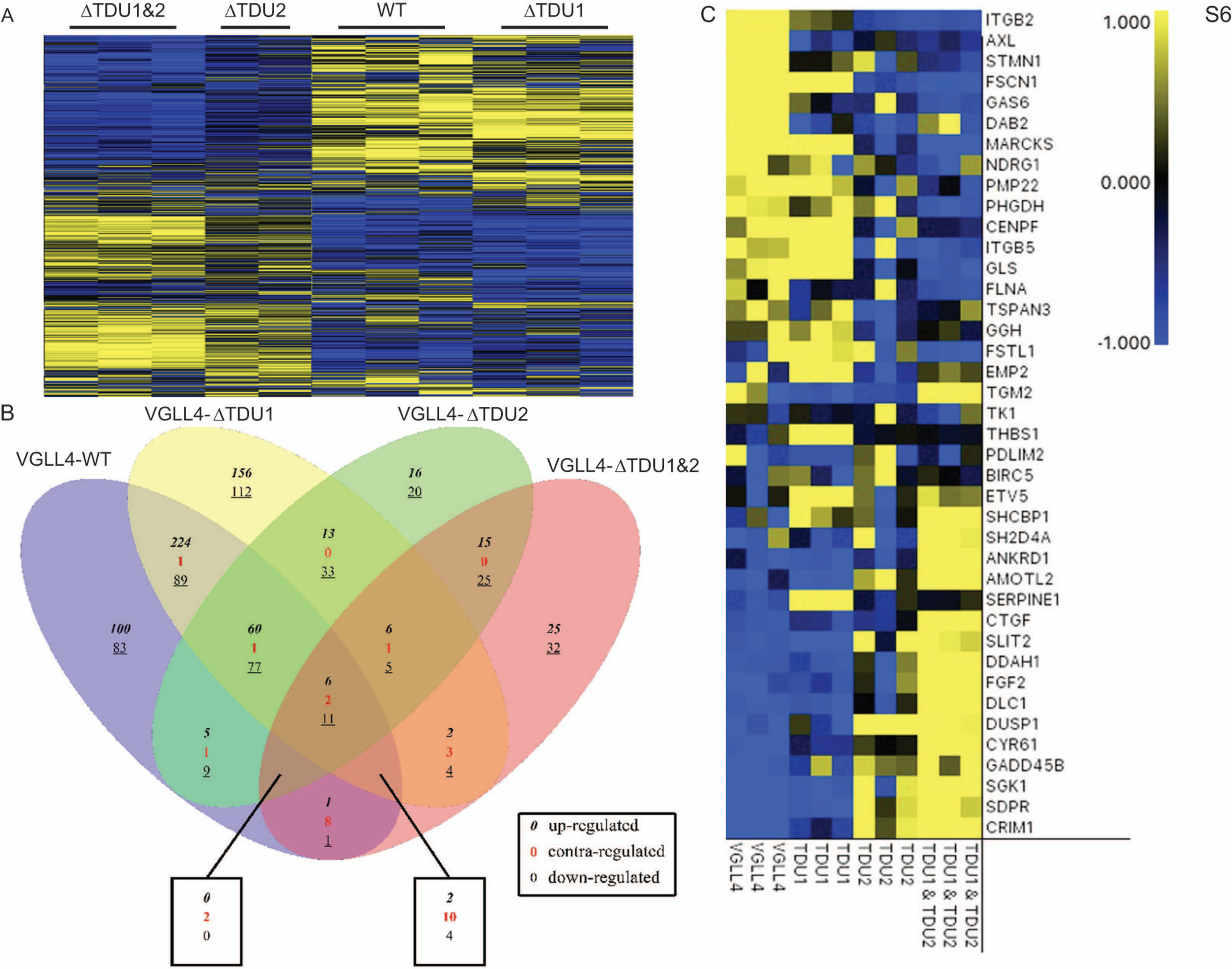
S6
A
ΔTDU1&2
ΔTDU2
WT
ΔTDU1
B
VGLL4-WT
VGLL4-ΔTDU1
VGLL4-ΔTDU2
VGLL4-ΔTDU1&2
156
112
16
20
224
1
89
13
0
33
15
0
25
100
83
60
1
77
6
1
5
25
32
6
2
11
5
1
9
2
3
4
1
8
1
0
2
0
2
10
4
0 up-regulated
0 contra-regulated
0 down-regulated
C
ITGB2
AXL
STMN1
FSCN1
GAS6
DAB2
MARCKS
NDRG1
PMP22
PHGDH
CENPF
ITGB5
GLS
FLNA
TSPAN3
GGH
FSTL1
EMP2
TGM2
TK1
THBS1
PDLIM2
BIRC5
ETV5
SHCBP1
SH2D4A
ANKRD1
AMOTL2
SERPINE1
CTGF
SLIT2
DDAH1
FGF2
DLC1
DUSP1
CYR61
GADD45B
SGK1
SDPR
CRIM1
1.000
0.000
-1.000
VGLL4
VGLL4
VGLL4
TDU1
TDU1
TDU1
TDU2
TDU2
TDU2
TDU1 & TDU2
TDU1 & TDU2
TDU1 & TDU2

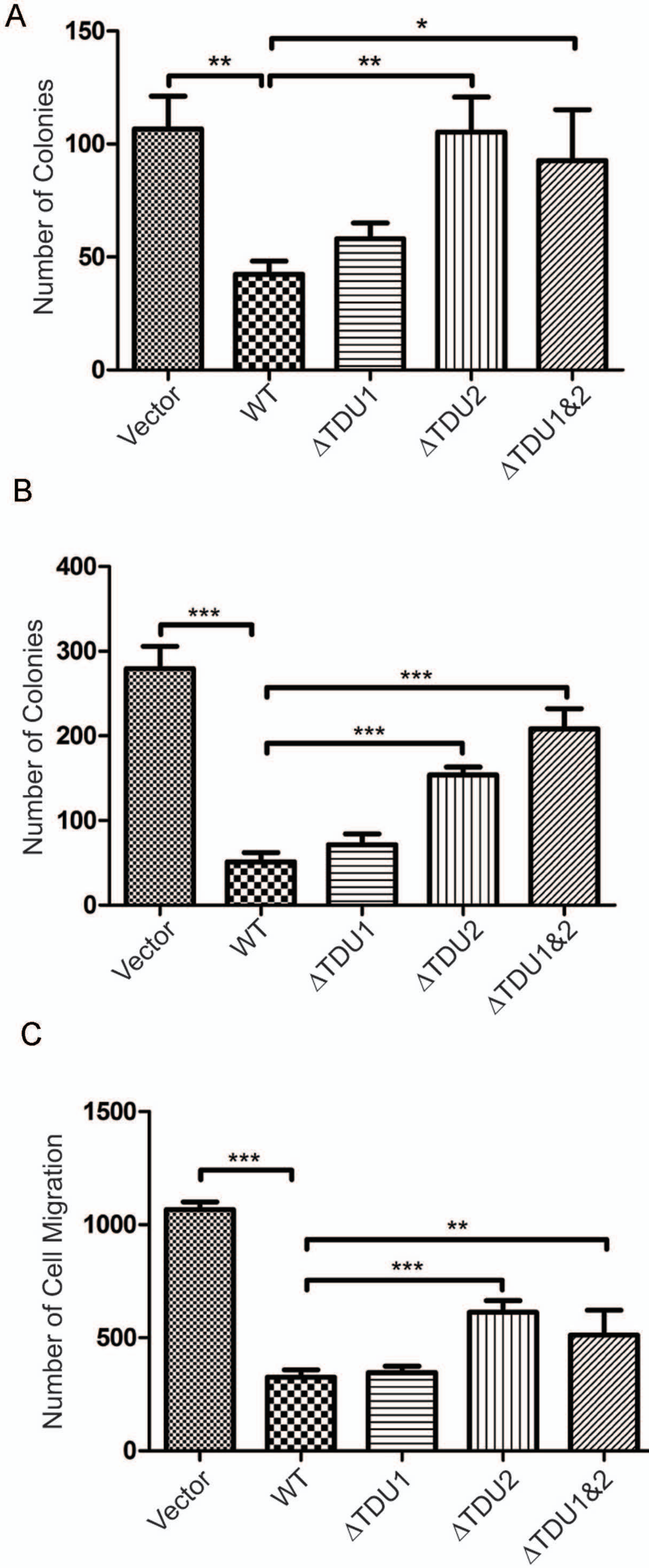
A
Number of Colonies
150
100
50
0
**
**
*
Vector
WT
ΔTDU1
ΔTDU2
ΔTDU1&2
B
Number of Colonies
400
300
200
100
0
***
***
***
Vector
WT
ΔTDU1
ΔTDU2
ΔTDU1&2
C
Number of Cell Migration
1500
1000
500
0
***
***
**
Vector
WT
ΔTDU1
ΔTDU2
ΔTDU1&2

Supplemental table 1. Univariate Cox Regression analysis of VGLL4 gene expression and breast cancer patient survival.

GO	SAMPLES		HAZARD RATIO	LCI (95%)	UCI (95%)	p VALUE
	VGLL4-High	VGLL4-Low				
GSE3143	79	78	0.44	0.23	0.81	0.009
GSE6130	17	16	0.05	0	0.78	0.032
GSE1456	80	79	0.57	0.36	0.91	0.018
GSE22226	64	64	0.45	0.25	0.81	0.008
